# The combined incremental prognostic value of left ventricular ejection fraction, late gadolinium enhancement and global circumferential strain assessed by cardiovascular magnetic resonance

**DOI:** 10.1186/1532-429X-17-S1-Q50

**Published:** 2015-02-03

**Authors:** Ify Mordi, Hiram Bezerra, David Carrick, Nikolaos Tzemos

**Affiliations:** 1 Institute of Cardiovascular and Medical Sciences, University of Glasgow, Glasgow, UK; 2 University Hospitals Case Medical Center, Case Western Reserve University, Cleveland, OH, USA

## Background

Left ventricular ejection fraction (LVEF) is powerful predictor of mortality and is used to guide treatment decisions. It is however subject to limitations, particularly when measured using echocardiography, as is most commonly done. Additionally, many major cardiovascular events occur in patients typically adjudged to be at lower risk using echocardiography (LVEF >35%). Assessment of myocardial deformation (strain) using tagging has the potential to overcome some of the limitations of LVEF. The value of global circumferential strain (GCS) measured by CMR tagging in patients with suspected cardiac disease has not been fully explored despite it being considered as the non-invasive gold standard method of assessment of LV deformation. We aimed to assess the incremental prognostic value of GCS measured using tagging for the prediction of major adverse cardiovascular events in addition to baseline clinical characteristics, LVEF and late gadolinium enhancement (LGE) in an unselected cohort of patients.

## Methods

We prospectively evaluated 539 consecutive patients referred for CMR who underwent a CMR protocol including cine imaging, tagging, and LGE. The combined primary endpoint was cardiovascular death, heart failure hospitalization and sustained ventricular arrhythmia requiring hospitalization or defibrillator therapy.

## Results

58 patients suffered the primary outcome over the mean follow-up period of 2.2 years. History of pre-existent ischemic heart disease and beta-blocker use were both significant clinical predictors of adverse outcome. All 3 CMR parameters were significant multivariable predictors of the primary outcome when added to significant clinical predictors (LVEF: HR 0.97; 95% CI 0.94-0.99, p=0.01; presence of LGE: HR 2.12; 95% CI 1.03-4.37, p=0.041; GCS: HR 1.10; 95% CI 1.02-1.20, p=0.019). Global χ^2^ increased significantly with the addition of both LGE and GCS. The optimal cut-off point for prediction of adverse outcome using GCS was -12.1%. Both the presence of LGE and GCS <-12.1% had independent prognostic value in the whole cohort and in patients with LVEF ≥35%. Additionally, patients with LVEF >35%, LGE present and reduced GCS (<-12.1%) had an equivalently poor prognosis to patients with LVEF <35%.

## Conclusions

We found in a large cohort of patients that GCS has incremental independent prognostic value in addition to clinical variables, LVEF and LGE. Furthermore, patients with LVEF >35% but reduced strain and LGE present have an equivalently poor prognosis to those with LVEF <35% and may benefit from more intensive life-saving therapy. The additional use of tagging and LGE may provide further risk stratification of patients to help guide management.

## Funding

N/A.

**Figure 1:**
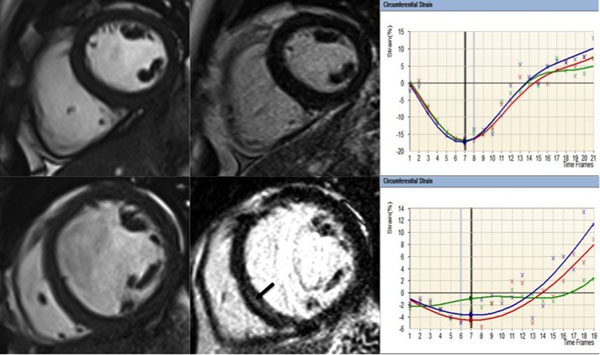
An example of the protocol with cine imaging, LGE and GCS in a normal patient (top) and a patient with severe LVSD (bottom).

**Figure 2:**
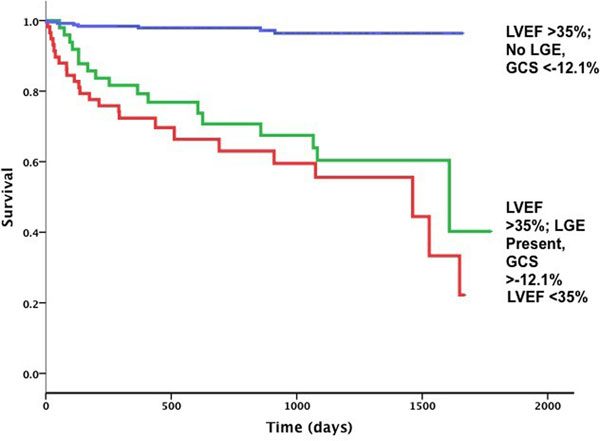
Kaplan-Meier curves. Patients with LVEF >35%, LGE present and reduced strain had an equivalently poor prognosis to patients with LVEF <35%.

